# The role of exosomal lncRNAs in cancer biology and clinical management

**DOI:** 10.1038/s12276-021-00699-4

**Published:** 2021-11-24

**Authors:** Wuwen Zhang, Qinshi Wang, Yi Yang, Siyuan Zhou, Ping Zhang, Tongbao Feng

**Affiliations:** Department of Clinical Laboratory, The Affiliated Changzhou No. 2 People’s Hospital of Nanjing Medical University, Changzhou, PR China

**Keywords:** Cancer, Molecular biology

## Abstract

Exosomes play a vital role in cell–cell communication within the cancer microenvironment. Exosomal long noncoding RNAs (lncRNAs) are important regulators in cancer development and are involved in multiple processes, including cancer cell proliferation, angiogenesis, metastasis, drug resistance, and immunomodulation. Changes in the levels of exosomal lncRNAs often appear with the occurrence and development of cancer. Therefore, exosomal lncRNAs can be used as biomarkers for cancer diagnosis and prognosis. Exosomal lncRNAs can also indicate the treatment response of patients receiving chemotherapy. Moreover, exosomal lncRNAs are potential therapeutic targets for cancer treatment. In this review, we summarize the role of exosomal lncRNAs in cancer biology as well as in clinical management. A more comprehensive and in-depth understanding of the role of exosomal lncRNAs in cancer may help us better understand the mechanism of cancer development and clinically manage cancer patients.

## Cancer: eavesdropping on communicating cancer cells

Cancer cells routinely release tiny membrane vesicles laden with RNAs that help promote tumor progression, but which could also offer useful biomarkers for diagnosis and reveal new drug targets. Many cells secrete such vesicles, known as exosomes, as a mode of intercellular communication. Researchers led by Tongbao Feng at Nanjing Medical University, Changzhou, China, have reviewed the evidence linking exosome-borne RNAs to tumor cell function. The authors focus on a class of molecules known as ‘long noncoding RNAs’ (lncRNAs), and show that exosomal lncRNAs are relayed as messages between tumor cells, promoting proliferation, metastatic spread, and acquisition of drug resistance. These RNAs can therefore also offer a useful window into the progress of disease and interventions that block such exosomally transmitted messages, and could potentially inhibit malignant growth and spread.

## Introduction

Exosomes are a group of extracellular vesicles with a diameter of 30–150 nm^1^. The release of exosomes is ubiquitous in a variety of cells in the human body. Exosomes are not simply fragments of cells; they carry abundant nucleic acids, proteins, and carbohydrates derived from their parent cells. Exosomes have been thought to be important messengers in cell–cell communication by transferring biological molecules to recipient cells, which can affect the activity and function of recipient cells^2^.

Cancer is still the main cause of death among humans. Recently, the role of exosomes in cancer development has gained increased attention because it has provided new insight in the study and management of cancers. Cancer cells have been thought to produce more exosomes than normal cells^3^. Cancer-derived exosomes are intentionally released by cancer cells and are important drivers of cancer cell functions. In addition, other cells in the cancer microenvironment, such as immune cells and stromal cells, are capable of releasing exosomes. Interactions between cancer cells and both immune cells and stromal cells mediated by exosomes play an essential role in cancer development.

The expression of noncoding RNAs is often found to undergo significant changes in cancer, which indicates that these noncoding RNAs are closely associated with cancer development^4^. Noncoding RNAs, including miRNAs and long noncoding RNAs (lncRNAs), can stably exist in exosomes, and noncoding RNAs carried by exosomes are also essential for cancer regulation^5^. Currently, related studies mainly focus on the value of exosomal miRNAs in cancers. Exosomal lncRNAs may also play an important role in cancer development. However, the value of exosomal lncRNAs in cancers has not yet been fully revealed. Existing studies have shown that exosomal lncRNAs not only are involved in cancer progression but also can be used as biomarkers for cancer diagnosis and treatment. In this review, we highlight the latest studies to summarize the role of exosomal lncRNAs in both cancer biology and clinical management.

## The crosstalk pattern mediated by exosomes in the cancer microenvironment

The cell components in the cancer microenvironment mainly include cancer cells, immune cells, fibroblast cells, and vessel endothelial cells. Exosomes are thought to play an important role in the crosstalk among these different cells. In the cancer microenvironment, cancer cells can communicate extensively with other cells via exosomes. First, cancer cells can influence the characteristics and biological behaviors of other cancer cells by transferring exosomes. In hepatocellular carcinoma (HCC), highly metastatic cancer cells can promote epithelial–mesenchymal transition (EMT) and metastasis of low-metastatic cancer cells by transferring exosomal miR-92a-3p and regulating the PTEN/Akt pathway^6^. In addition, cancer cells can act on immune cells by delivering exosomes. In breast cancer, cancer cell-derived exosomal lncRNA SNHG16 can upregulate the expression of CD73 on Vδ1 Treg cells through the SNHG16/miR-16-5p/SMAD5 regulatory axis^7^. In addition, cancer cells can interact with stromal cells with exosomes as intercellular mediators. For example, bladder cancer-derived exosomes induce the transformation of fibroblasts into cancer-associated fibroblasts through TGF-β signaling pathways^8^. Gastric cancer (GC)-derived exosomes can deliver miR-130a into vascular endothelial cells and consequently promote angiogenesis^9^. On the other hand, exosomes from immune cells and stromal cells can reverse the phenotype and biological behaviors of cancer cells. For example, exosomes from tumor-associated macrophages (TAMs) can transmit HIF-1α-stabilizing long noncoding RNA (HISLA) to breast cancer cells, thus enhancing aerobic glycolysis and apoptosis resistance of breast cancer cells^10^. Exosomes from cancer-associated fibroblasts have been discovered to promote colorectal cancer (CRC) metastasis and chemoresistance by delivering miR-92a-3p to CRC cells^11^. Additionally, immune cells can interact with other immune cells and endothelial cells. In epithelial ovarian cancer (EOC), TAM-derived exosomes can transmit miR-29a-3p and miR-21-5p to CD4+ T cells and suppress STAT3 expression, which induces an imbalance of Treg/Th17 cells^12^. TAM-derived exosomes can also regulate endothelial cells in EOC. Exosomal miR-146b-5p from TAMs suppresses the migration of human vessel endothelial cells through the TRAF6/NF-κB/MMP2 pathway. Interestingly, the suppressive effect can be reversed by exosomal lncRNAs from EOC^13^. In summary, exosome-mediated interactions are widely prevalent in different cell types within the cancer microenvironment. Regulatory molecules such as lncRNAs, miRNAs, and proteins carried by exosomes play an important role in altering the phenotypes and biological behaviors of recipient cells, which is significantly associated with cancer progression.

### The role of exosomal lncRNAs in cancer biology

**Fig. 1:**
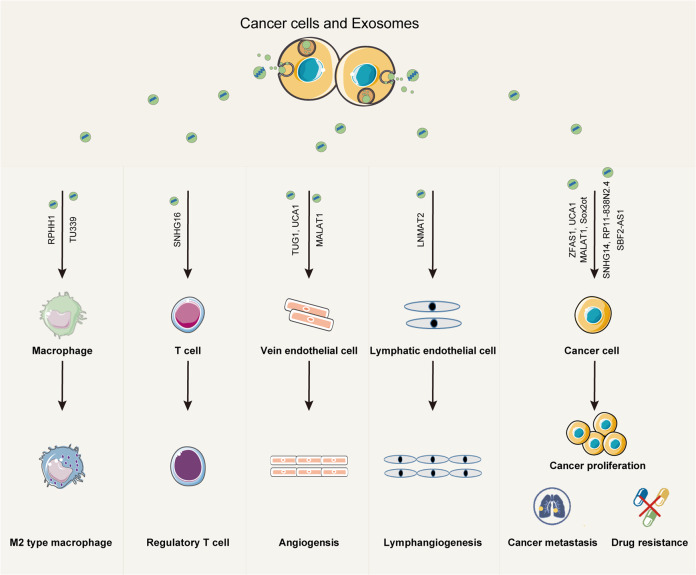
The role of exosomal lncRNAs in cancer biology. Exosomes play an important role in mediating the interaction between cancer cells and both immune cells and stromal cells within the cancer microenvironment. Exosomal lncRNAs from cancer cells can promote immune modulation, angiogenesis, cancer proliferation, metastasis, and drug resistance.

In recent years, the roles of exosomal lncRNAs in cancer development have gained increasing attention. Exosomal lncRNAs are involved in multiple important steps in cancer development, including cancer cell proliferation, angiogenesis, invasion and metastasis, drug resistance, and immune modulation (Fig. 1). In this section, we will further elaborate on the role of exosomal lncRNAs in different processes of cancer development.

### Exosomal lncRNAs and cancer cell proliferation

Cancer cell proliferation is the basic step in cancer progression. Exosomal lncRNAs in the cancer microenvironment are found to promote this process. For example, in esophageal squamous cell carcinoma (ESCC), lncRNA ZFAS1 is elevated in cancer tissues and can be transmitted among cancer cells via exosomes. Exosomal ZFAS1 can competitively inhibit miR-124 expression and thereby upregulate the expression of STAT3 protein. Through the regulation of the miR-124/STAT3 axis, exosomal ZFAS1 can promote ESCC cell proliferation and inhibit apoptosis. Moreover, in vivo experiments show that exosomal ZFAS1 can promote ESCC tissue proliferation in tumor-bearing mice^14^. LncRNA ZFAS1 is also upregulated in GC tissues and cells. ZFAS1 knockdown can suppress cancer cell cycle progression, induce apoptosis, and inhibit EMT, while high levels of exosomal ZFAS1 can promote GC cell proliferation and migration^15^. Hypoxia is often present in the cancer microenvironment and can promote cancer progression. In bladder cancer under hypoxic conditions, the bladder cancer cell line 5637 can release more exosomes containing lncRNA UCA1. Exosomal UCA1 can be internalized by other bladder cancer cell lines and promote cell proliferation through EMT in vitro and in vivo^16^. Together, exosomal lncRNAs from cancer cells or other cells in the cancer microenvironment can be delivered to recipient cancer cells. With the activation of related signaling pathways and changes in cell phenotypes, the proliferation potential of cancer cells is enhanced.

### Exosomal lncRNAs and angiogenesis in cancer

Angiogenesis plays a key role in cancer development. Noncoding RNAs are associated with cancer angiogenesis by acting on vascular endothelial cells. Recently, lncRNAs from cancer exosomes were found to promote angiogenesis by acting on human umbilical vein endothelial cells (HUVECs) in cancers. In EOC, lncRNA metastasis-associated lung adenocarcinoma transcript 1 (MALAT1) from cancer cells can be transferred to HUVECs via exosomes. Exosomal MALAT1 was demonstrated to promote angiogenesis in both in vitro and in vivo assays. A study on the mechanism of exosomal MALAT1 activity revealed that it can possibly stimulate the expression of angiogenesis-related genes, including VEGF-A, VEGF-D, and angiogenin, thereby facilitating cancer angiogenesis^17^. Similarly, lncRNA taurine upregulated 1 (TUG1) is elevated in cervical cancer cells and can be delivered to HUVECs via exosomes. Exosomal TUG1 can suppress caspase-3 activity and impact apoptosis-related proteins to promote HUVEC proliferation, thus promoting angiogenesis^18^. Exosomal lncRNAs can act on miRNAs to activate downstream pathways that participate in angiogenesis. In pancreatic cancer, cancer cells under hypoxic conditions can release high levels of exosomes carrying lncRNA UCA1. When internalized by HUVECs, exosomal UCA1 from hypoxic pancreatic cancer cells can inhibit miR-96-5p, upregulate the expression of its target gene AMOTL2, and subsequently activate the ERK signaling pathway, which promotes the migration and tube formation of HUVECs^19^. Collectively, exosomal lncRNAs derived from cancer cells can act on vascular endothelial cells to promote angiogenesis by stimulating the expression of angiogenesis-related proteins or inhibiting the function of antiangiogenic miRNAs. However, the exact mechanism underlying this process needs to be elucidated with further research.

### Exosomal lncRNAs and cancer metastasis

Exosomal lncRNAs can promote cancer invasion or metastasis by targeting cancer cells or cancer-promoting cells. For example, crosstalk between cancer cells and lymphatic endothelial cells mediated by exosomal lncRNAs can promote lymph node metastasis. In bladder cancer with VEGF-C-independent lymph node metastasis, lncRNA lymph node metastasis-associated transcript 2 (LNMAT2) is enriched in bladder cancer cell-derived exosomes. When taken up by human lymphatic endothelial cells (HLECs), exosomal LNMAT2 can increase prospero homeobox 1 (PROX1) expression by recruiting hnRNPA2B1 and enhancing H3K4 trimethylation in the PROX1 promoter and ultimately promote lymphangiogenesis and lymphatic metastasis^20^. Exosomal lncRNAs from metastatic cancer cells can also act on primary cancer cells and contribute to metastasis. In patients with CRC, lncRNA MALAT1 is upregulated in both metastatic CRC cells and their exosomes. Exosomal MALAT1 can increase FUT4 expression in primary CRC cells by sponging miR-26a/26b. Furthermore, upregulated FUT4 was demonstrated to enhance fucosylation levels and activate the PI3K/AKT/mTOR pathway in in vitro and in vivo assays, which finally resulted in the invasion and metastasis of CRC^21^. Likewise, in pancreatic ductal adenocarcinoma (PDAC), exosomal lncRNA Sox2ot derived from highly invasive PDAC cells can be delivered and taken up by less invasive PDAC cells. Exosome-mediated transfer of lncRNA Sox2ot can competitively bind to the miR-200 family to regulate the expression of Sox2, which promotes the EMT process and induces stem cell-like properties in recipient cells. Ultimately, these phenotypic alterations make recipient cells more invasive and contribute to cancer metastasis^22^.

### Exosomal lncRNAs and drug resistance in cancer

Drug resistance in cancer is a crucial reason for the poor outcome of cancer patients. The mechanisms underlying cancer drug resistance are largely unclear. Recently, exosomes have been found to deliver specific lncRNAs to cancer cells and contribute to drug resistance. In breast cancer, cancer cells resistant to trastuzumab can release high levels of exosomes containing lncRNA SNHG14 and transmit the lncRNA to drug-sensitive cancer cells. Exosomal SNHG14 uptake by recipient cells is likely to activate the Bcl-2/Bax signaling pathway and inhibit apoptosis. Subsequently, these drug-sensitive cancer cells gained trastuzumab resistance from resistant cancer cells^23^. A similar phenomenon has also been found in the resistance of non-small-cell lung cancer (NSCLC) cells to erlotinib. LncRNA RP11-838N2.4 levels were higher in NSCLC cells resistant to erlotinib than in NSCLC cells without erlotinib resistance. Functional experiments showed that the knockdown of RP11-838N2.4 could promote erlotinib-induced cytotoxicity. Moreover, exosomes loaded with RP11-838N2.4 can be taken up by NSCLC cells sensitive to erlotinib and induce erlotinib resistance^24^. However, the mechanism underlying the transmission of erlotinib resistance by exosomal RP11-838N2.4 needs further research. Exosomal lncRNAs can act as ceRNAs to miRNAs and promote drug resistance in cancer. In glioblastoma tissues and cells with temozolomide resistance, lncRNA SBF2-AS1 is overexpressed and can be secreted by exosomes. LncRNA SBF2-AS1 can sponge miR-151a-3p, which relieves the suppression to its target, X-ray repair cross-complementing 4, thus enhancing DNA double-strand break repair in glioblastoma cells, which reportedly promotes temozolomide resistance^25^. In vitro and in vivo assays showed that exosomal SBF2-AS1 from temozolomide-resistant glioblastoma cells can accelerate DNA damage repair in recipient cells and enhance chemoresistance to temozolomide^26^.

### Exosomal lncRNAs and immunomodulation of cancer

Exosomal lncRNAs play an important role in the interaction between cancer cells and immune cells. Thus, exosomal lncRNAs are involved in cancer immunomodulation. In HCC, cancer cell-derived exosomes can transmit lncRNA TU339 to macrophages. The increased expression of lncRNA TU339 polarizes macrophages toward an anti-inflammatory phenotype with reduced proinflammatory cytokine production, decreased costimulatory molecule expression, and compromised phagocytosis. This polarization is probably associated with HCC progression^27^. Similarly, CRC cell-derived exosomal lncRNA RPPH1 can be delivered to macrophages and mediate M2 polarization of macrophages. M2 macrophages are tumorigenic and can promote CRC cell proliferation and metastasis^28^. In addition, exosomal lncRNAs are involved in the establishment of an immunosuppressive cancer microenvironment. In breast cancer, lncRNA SNHG16 can be transmitted to Vδ1 T cells via exosomes. Exosomal SNHG16 can sponge miR-16-5p as a ceRNA and result in the derepression of its target gene SMAD5. Subsequently, the TGF-β/SMAD5 pathway is activated, which induces upregulated expression of CD73 in Vδ1 T cells. CD73 + Vδ1 T cells are a group of regulatory T cells that exert immunosuppressive functions in the breast-cancer microenvironment^7^.

### The value of exosomal lncRNAs in the clinical management of cancer

Exosome-mediated delivery of lncRNAs to recipient cells in the cancer microenvironment is widely involved in multiple processes of cancer development. Therefore, alterations in exosomal lncRNA levels are indicative of cancer diagnosis, prognosis and medical treatment. Since exosomes can enter the peripheral blood through small vessels, exosomal lncRNAs in circulation could serve as potential biomarkers for the clinical management of cancer. We have summarized the clinical value of exosomal lncRNAs in cancer in Table 1.

**Table 1:** The value of exosomal lncRNAs in the clinical management of cancer.

Cancer type	Value	lncRNA	Reference
Gastric cancer	Cancer diagnosis	UEGC1	Ref. ^29^
Colorectal cancer	Cancer diagnosis	CRNDE-h	Ref. ^30^
Non-small-cell lung cancer	Cancer diagnosis	GAS5	Ref. ^31^
Epithelial ovarian cancer	Prognosis evaluation	aHIF	Ref. ^32^
Colorectal cancer	Prognosis evaluation	91H	Ref. ^33^
Hepatocellular carcinoma	Prognosis evaluation	ATB	Ref. ^34^
Bladder cancer	Therapeutic target	LNMAT2	Ref. ^20^
Renal cancer	Therapeutic target	lncARSR	Ref. ^35^
Colorectal cancer	Treatment efficacy	UCA1	Ref. ^36^

### Exosomal lncRNAs and cancer diagnosis

To develop a noninvasive diagnostic method for early-stage gastric cancer (EGC), researchers used RNA-sequencing technology to screen out highly expressed lncRNA profiles in circulating exosomes from EGC patients and further determined that lncRNA UEGC1 is a specific exosomal lncRNA in EGC. LncRNA UEGC1 is remarkably upregulated in exosomes from EGC patients and in GC cells. Moreover, exosomal UEGC1 can significantly distinguish EGC patients from chronic atrophic gastritis patients and healthy people, with AUC values of 0.8406 and 0.8760, respectively^29^. Likewise, in patients with CRC, exosomal lncRNA CRNDE-h was found to be elevated compared to that in benign colorectal disease patients and healthy people. Receiver operating characteristic (ROC) curves were established to evaluate the ability of exosomal CRNDE-h to distinguish CRC patients, benign colorectal disease patients, and healthy people. When the clinical value was set at 0.020, the AUC reached 0.892, and the sensitivity and specificity for diagnosing CRC were 70.3% and 94.4%, respectively. The diagnostic performance is better than that of the traditional marker CEA^30^. In addition, downregulated exosomal lncRNAs can be applied in diagnosing cancer. The level of exosomal lncRNA GAS5 was found to be lower in NSCLC patients than in healthy controls. Exosomal GAS5 can significantly distinguish stage I NSCLC patients and healthy people with an AUC of 0.822 by ROC analysis^31^. In summary, alterations in exosomal lncRNA levels are of diagnostic value for multiple cancers. Exosomal lncRNAs are also promising cancer diagnostic biomarkers because they may provide more information than traditional biomarkers.

### Exosomal lncRNAs and cancer prognosis

Exosome-based biomarkers have gained increasing attention for determining cancer prognosis. For example, proteins and miRNAs in exosomes are often recommended as prognostic biomarkers. Recently, studies have gradually focused on the role of exosomal lncRNAs in cancer prognosis. In EOC, the level of serum exosomal lncRNA aHIF is upregulated and positively correlated with the level of aHIF in cancer tissues. A Cox multivariate regression model revealed that serum exosomal aHIF levels were an independent prognostic factor of EOC. Kaplan-Meier analysis suggested that EOC patients with higher levels of exosomal aHIF had poorer overall survival^32^. In patients with CRC, exosomal lncRNA 91H can enhance CRC metastasis by modifying HNRNPK. Thus, exosomal 91H might serve as a plasma-based biomarker for CRC prognosis. CRC patients with high exosomal 91H also have a higher risk of CRC metastasis and recurrence than patients with low exosomal 91H^33^. In addition, researchers evaluated the prognostic value of exosomal lncRNA ATB in patients with HCC. The level of exosomal ATB was associated with tumor stage and the presence of portal vein thrombosis. The overall survival and progression-free survival of HCC patients with higher levels of exosomal ATB are significantly shortened^34^. However, studies on the use of exosomal lncRNAs as prognostic markers are insufficient. More research is needed to explore the prognostic value of exosomal lncRNAs so that they can be implemented for clinical management of cancer.

### Exosomal lncRNAs and cancer therapy

Since exosomal lncRNAs can promote cancer development or convey drug resistance among cancer cells, exosomal lncRNAs are potential therapeutic targets for cancer treatment. Developing drugs to inhibit the transfer of exosomal lncRNAs between drug-resistant cells and drug-sensitive cells may improve therapeutic efficacy and prevent cancer progression. LNMAT2 is an upregulated lncRNA in bladder cancer patients with lymph node metastasis. Exosomal LNMAT2 from bladder cancer cells can be internalized by HLECs and promote lymphatic metastasis. An in vitro study showed that exosomes from LNMAT2-silenced cells could inhibit HLEC tube formation and migration. The results indicated that decreased LNMAT2 expression could prevent lymph node metastasis and that LNMAT2 might be a therapeutic target for bladder cancer with lymph node metastasis^20^. In renal cancer, lncARSR functions as an oncogene, and exosomal lncARSR can transfer sunitinib resistance from sunitinib-resistant cells to sunitinib-sensitive cells. Researchers established a nude mouse model of renal cancer with sunitinib resistance by xenografting renal cancer cells or patient-derived xenografts. When treated with locked nucleic acids (LNAs) targeting lncARSR, the xenografts showed reduced expression of lncARSR and restored responsiveness to sunitinib. Thus, lncARSR can serve as a therapeutic target for renal cancer with sunitinib resistance^35^. Exosomal lncRNAs are also promising biomarkers for guiding drug therapy, as the level of exosomal lncRNAs is associated with drug treatment responses in cancer patients. For example, in CRC patients receiving cetuximab, patients who did not respond to the treatment had higher levels of exosomal UCA1 than did patients who showed a partial or complete response. Thus, the detection of exosomal UCA1 can be used to predict the treatment response of CRC patients receiving cetuximab^36^.

## Discussion and perspectives

The membrane structure of exosomes can protect lncRNAs, miRNAs, and proteins from degradation; thus, exosomes are important biological molecule carriers and play an essential role in intercellular communication. In the body fluids of cancer patients, exosomes are mainly derived from cancer cells. The exploration of exosomes provides us with new insight into cancer biology, and it helps us develop new cancer diagnosis and treatment strategies.

Exosomal lncRNAs are involved in multiple processes of cancer development, including cancer cell proliferation, angiogenesis, metastasis, drug resistance, and immunomodulation. Capturing important biological information from exosomal lncRNAs can help us better understand and manage cancers. The levels of cancer-related exosomal lncRNAs are associated with the severity of disease in cancer patients; thus, exosomal lncRNAs are potential cancer biomarkers. As an emerging class of cancer biomarker, exosomal lncRNAs have advantages over traditional markers. In addition to their role in cancer diagnosis and prognosis, exosomal lncRNAs can be used as treatment targets. Since exosomal lncRNAs play a crucial role in cancer progression and the spread of drug resistance, drugs that inhibit the function of exosomal lncRNAs may be effective in cancer therapy. At present, some studies have partially elucidated the value of exosomal lncRNAs in cancer. Nevertheless, the contribution of exosomal lncRNAs in cancer needs further exploration, and exosomal lncRNAs are expected to play a more important role in the clinical management of cancer.
